# Dietary Bioactive Compounds: Cellular Sensory Pathways, Microbial Partners, and Clinical Realities

**DOI:** 10.3390/nu18183001

**Published:** 2026-09-14

**Authors:** Elandia Aparecida dos Santos, Jacqueline Isaura Alvarez-Leite

**Affiliations:** Department of Biochemistry and Immunology, Institute of Biological Sciences, Federal University of Minas Gerais (UFMG), Avenida Presidente Antônio Carlos, 6627, Pampulha, Belo Horizonte 31270-901, MG, Brazil; elandianutri@gmail.com

**Keywords:** bioactive compounds, polyphenols, gut microbiota, inflammation, oxidative stress, chronic diseases, bioavailability, precision nutrition, metabolic regulation

## Abstract

**Background/Objectives:** Dietary bioactive compounds (BCs) modulate multiple cellular pathways involved in inflammation, oxidative stress, energy metabolism, and tissue homeostasis. This narrative review aimed to synthesize the mechanistic roles and therapeutic relevance of major dietary BC classes, including polyphenols, carotenoids, organosulfur compounds, and polyunsaturated fatty acids, in chronic non-communicable diseases. **Methods:** We conducted a narrative review to integrate current evidence on the classification, bioavailability, molecular targets, microbiota interactions, and clinical relevance of dietary BCs. The literature considered experimental, observational, and clinical studies addressing key signaling pathways, including NF-κB, Nrf2, AMPK, mTOR, SIRT1, PPARs, and the NLRP3 inflammasome, as well as the diet–microbiota–host axis. **Results:** The literature indicates that BCs exert pleiotropic effects through the coordinated modulation of transcription factors, metabolic sensors, nuclear receptors, and inflammatory mediators. Their biological activity is also shaped by bioavailability, food matrix effects, host metabolism, and gut microbial transformation into bioactive metabolites. Across obesity, type 2 diabetes, cardiovascular disease, inflammatory bowel disease, and neurodegenerative conditions, BCs appear associated with improved inflammatory, metabolic, and redox regulation. However, findings remain limited by heterogeneity in study design, intervention duration, and compound characterization. **Conclusions:** Dietary BCs show therapeutic potential as modulators of molecular and microbial pathways relevant to chronic diseases. However, greater standardization in study design and longer-term human trials are needed to support robust evidence-based recommendations.

## 1. Introduction

Although genetic factors contribute to individual susceptibility, growing evidence shows that environmental factors, especially diet, play a determining role in both the prevention and progression of chronic non-communicable diseases (NCDs) [1,2,3,4].

In this context, nutrition is no longer understood only as a source of energy and essential nutrients. It is now recognized as an important modulator of physiological, metabolic, and immunological processes [5,6].

In parallel with this paradigm shift, dietary bioactive compounds (BC) in food have received increasing attention from the scientific community because they can interact with multiple molecular targets involved in maintaining cellular homeostasis [4,7,8]. Unlike classic nutrients, BC are not considered essential for survival, but they exert relevant biological effects that can influence various body functions [9].

These compounds comprise a structurally heterogeneous group, including polyphenols, flavonoids, carotenoids, organosulfur compounds, curcuminoids, capsaicinoids, terpenes, polyunsaturated fatty acids, and other natural metabolites present in fruits, vegetables, spices, whole grains, oilseeds, teas, cocoa, fish, and bee products [10,11].

Advances in molecular biology, metabolomics, and nutrigenomics have shown that many of these compounds modulate cell signaling pathways, regulating processes related to inflammation, oxidative stress, energy metabolism, immune response, mitochondrial function, and gut barrier integrity [11,12].

Among the main molecular targets are transcription factors, nuclear receptors, and signaling proteins, such as nuclear factor kappa B (NF-κB), the nuclear factor erythroid 2-related factor 2 (Nrf2), the AMP-activated protein kinase (AMPK), the *Mechanistic* target of rapamycin (mTOR), the Sirtuin 1 (SIRT1), the proliferator-activated receptors (PPARs), and the NLR family pyrin domain containing 3 (NLRP3) inflammasome. Coordinated modulation of these pathways may reduce chronic low-grade inflammation, strengthen endogenous antioxidant systems, improve metabolic function, and preserve tissue homeostasis [13,14,15].

Another aspect of BC that has drawn increased interest is their bidirectional interactions with the gut microbiota. Many of these compounds reach the colon virtually intact, where the microbiota metabolize them into intestinal metabolites that are often more available and biologically active than the precursor molecules [16,17,18].

On the other hand, BCs can modulate microbiota composition and metabolic activity, favoring the growth of beneficial bacteria, increasing short-chain fatty acid production, reducing metabolic endotoxemia, and helping maintain intestinal barrier integrity. These mechanisms reinforce the importance of the diet–microbiota–host axis as a main mediator of the physiological effects attributed to dietary bioactives [9,19].

Despite the growing number of experimental, observational, and clinical studies involving BC, the literature still has limitations. Most reviews focus on specific compounds or isolated diseases, while few approaches integrate the different classes of bioactives from the perspective of their shared molecular mechanisms [20,21,22,23].

In addition, bioavailability, metabolism, interaction with the food matrix, interindividual variability of the microbiota, and the quality of clinical evidence remain challenges for translating basic knowledge into clinical practice [24,25,26,27].

Given this scenario, it is necessary to critically gather the available evidence, emphasizing not only the biological effects of BC, but also the cellular and molecular mechanisms responsible for their actions and their therapeutic potential in different clinical conditions. An integrated understanding of these processes can contribute to more effective nutritional strategies, inform evidence-based recommendations, and guide future research in precision nutrition.

This review aims to critically synthesize the current evidence on the main BC in the diet, addressing their classification, bioavailability, key molecular targets, biological activities, and therapeutic potential in the prevention and management of human diseases, and discussing translational challenges and future perspectives for clinical application. Search terms combined controlled vocabulary and free-text keywords for each bioactive-compound class (e.g., “polyphenols”, “carotenoids”, “organosulfur compounds”, “curcuminoids”, “capsaicinoids”, “polyunsaturated fatty acids”) combined with “gut microbiota”, “bioavailability”, “signaling pathway”, and “clinical trial”. Records were first screened by title and abstract, followed by full-text assessment of retained studies. Exclusion criteria comprised non-English-language publications, conference abstracts, and case reports. Additionally, articles were analyzed using the Consensus artificial intelligence platform, with filtering restricted to publications between 2010 and 2026 and classified within journal quartiles Q1–Q2, excluding preprints. We included experimental, observational, and clinical studies that addressed molecular mechanisms, bioavailability, or the clinical relevance of dietary bioactives. No formal quality assessment was performed, as this review aimed to provide a broad overview of the literature rather than a systematic evaluation.

### Bioactive Compounds in Diet: Concepts, Classification and Bioavailability

BC correspond to a broad and heterogeneous class of natural substances present in foods of plant and animal origin, capable of exerting relevant biological effects beyond their classic nutritional contribution [28,29,30,31].

Although they are not considered essential nutrients for survival or disease prevention, these substances actively participate in regulating cellular processes and metabolic and immunological functions, contributing to homeostasis and reducing the risk of chronic diseases [28,30,31]. Thus, they are among the components responsible for the health benefits attributed to dietary patterns rich in minimally processed foods [9,30].

Contrary to the widespread concept that certain foods are intrinsically functional, contemporary literature argues that functionality depends on demonstrating a measurable physiological effect in humans, obtained under conditions of habitual consumption and supported by consistent scientific evidence [31,32,33,34,35].

Beyond the biological definition of functionality, the translation of these findings into food and supplement claims is also shaped by the regulatory and commercial context in which bioactive compounds are marketed. Regulatory agencies such as the European Food Safety Authority (EFSA) and the U.S. Food and Drug Administration (FDA) require that health claims for functional foods and bioactive-compound-containing supplements be supported by robust human evidence of a specific, measurable physiological effect. These agencies generally distinguish between health claims made for whole foods and those made for isolated or concentrated compounds, the latter being subject to stricter substantiation requirements closer to those applied to nutraceuticals or pharmaceuticals. In practice, however, commercial “functional food” marketing frequently outpaces the underlying scientific evidence, extrapolating preclinical or mechanistic findings to consumer-facing claims before adequate human clinical validation is available—a gap that reinforces the need for critical appraisal of the evidence base discussed throughout this review.

**Figure 1:**
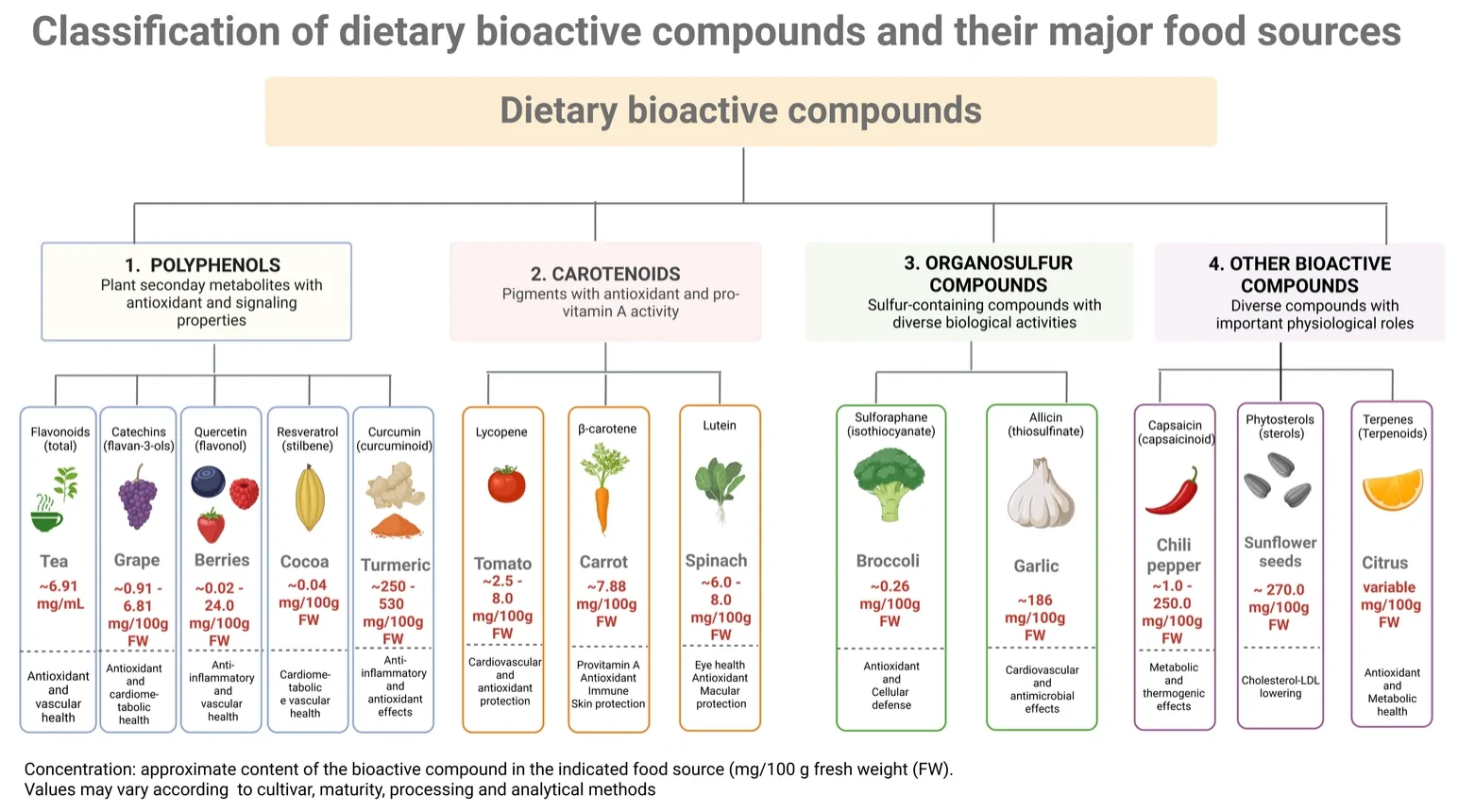
Classification of dietary bioactive compounds and representative food sources. Major classes of dietary bioactive compounds (BCs) are presented together with representative compounds, food sources, approximate concentration ranges, and selected biological or health-related effects. Reported concentrations are representative ranges and may vary substantially according to cultivar, variety, geographic origin, maturity, processing, storage conditions, and analytical methodology. The indicated health effects summarize major biological activities associated with the compounds or food sources and should not be interpreted as exclusive or clinically established effects of the foods shown. Abbreviations: BCs, bioactive compounds; FW, fresh weight; LDL, low-density lipoprotein.

Several classes of BC have been identified. Among the most studied are the polyphenols, comprising flavonoids, phenolic acids, stilbenes, and lignans; the carotenoids, represented by β-carotene, lycopene, lutein, and zeaxanthin; organosulfur compounds, present in cruciferous vegetables and species of the genus Allium; curcuminoids and capsaicinoids: found in turmeric (Curcuma longa) and peppers of the genus Capsicum, respectively; phytosterols, long-chain polyunsaturated fatty acids: such as eicosapentaenoic acid (EPA) and docosahexaenoic acid (DHA) [36,37,38]. These compounds are widely distributed in fruits, vegetables, legumes, whole grains, oilseeds, spices, aromatic herbs, cocoa, coffee, teas, olive oil, fish, and bee products [28,39,40] (Figure 1). The composition of each bioactive varies according to genetic factors, environmental conditions, stage of maturation, industrial processing, storage, and culinary preparation, aspects that directly influence their final concentration and potential physiological effect [6,30,41,42,43].

The magnitude of the BC physiological effects depends directly on its bioavailability. After ingestion, these compounds are released from the food matrix, absorbed in the gastrointestinal tract, and subjected to extensive intestinal and hepatic metabolic processes, including hydrolysis, oxidation, glucuronidation, sulfation, and methylation. As a result, only a small fraction of the original compound reaches systemic circulation; most is transformed into metabolites with biological properties distinct from the precursor molecule [44,45,46,47].

The gut microbiota also influences BC bioavailability. Several polyphenols reach the colon virtually intact, where gut bacteria metabolize them into lower-molecular-weight molecules that are often more bioavailable and biologically active [16,48,49,50]. Polyphenols also modulate the composition and metabolic activity of the microbiota, favoring the growth of beneficial microorganisms and increasing the production of metabolites, such as short-chain fatty acids [51,52,53]. This bidirectional interaction between BC and the microbiota is a key mechanism by which diet influences inflammatory, metabolic, and immunological processes, and it is a pillar of precision nutrition [54,55,56,57,58,59,60].

## 2. Molecular Targets and Cellular Mechanisms Involved in Health Promotion

Transcriptomics, proteomics, and metabolomics techniques have shown that the effects of BC do not result from acting on a single cellular target but from the coordinated modulation of multiple signaling pathways involved in maintaining homeostasis [20,35,61,62]. BC exert pleiotropic effects by simultaneously regulating transcription factors, protein kinases, nuclear receptors, and metabolic sensors [14,63,64,65].

**Figure 2:**
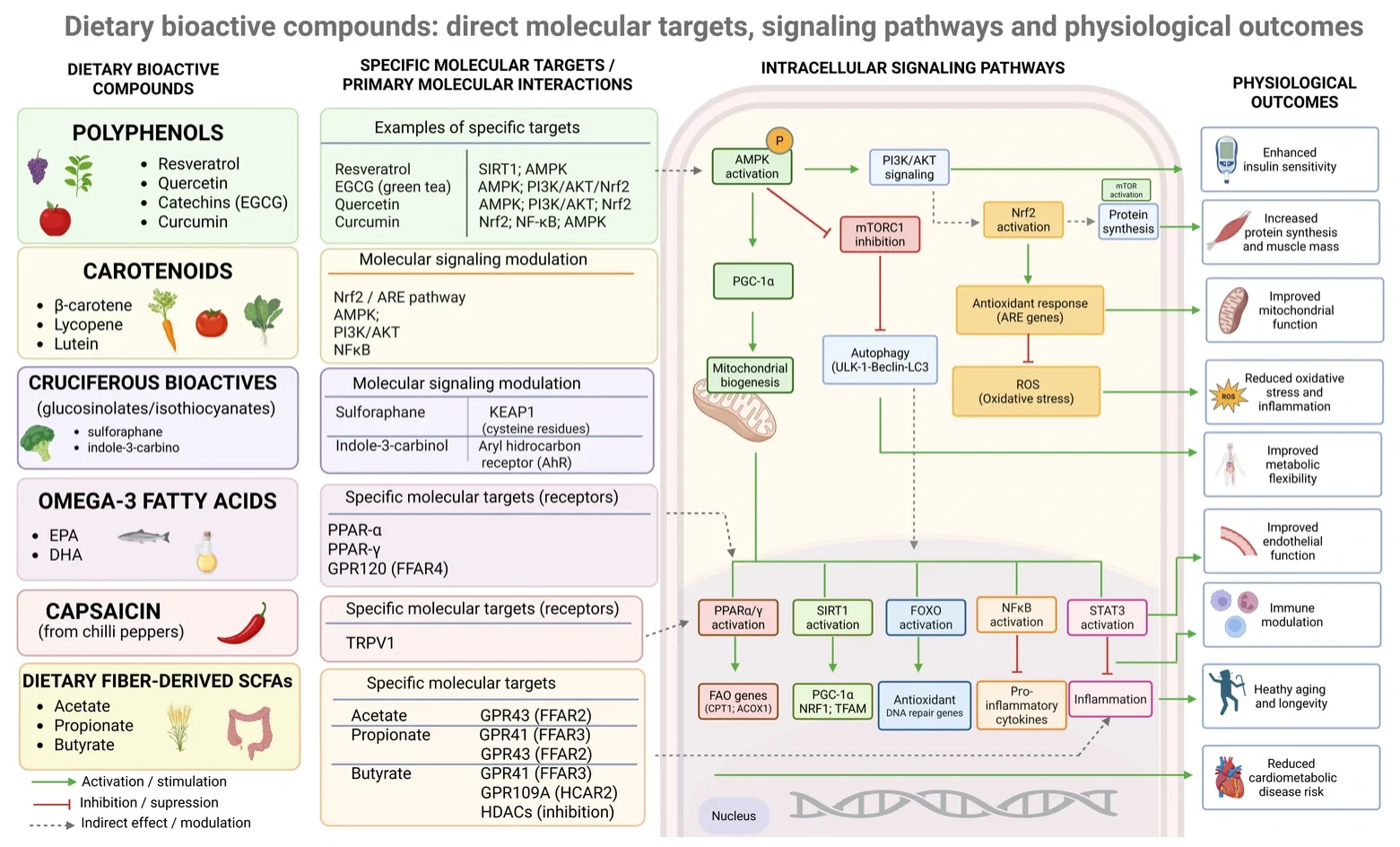
Dietary bioactive compounds: molecular targets, signaling pathways, and physiological outcomes. Representative classes (polyphenols, carotenoids, cruciferous bioactives, omega-3 fatty acids, capsaicin, and SCFAs) modulate key pathways including AMPK, PI3K/AKT, Nrf2/ARE, and NF-κB. Direct molecular interactions are distinguished from downstream modulation. Green arrows denote activation or stimulation; red symbols indicate inhibition or suppression; dashed arrows represent indirect effects. Depicted mechanisms are representative and biological relevance varies by specific compound, dose, and experimental context. Abbreviations: AhR, aryl hydrocarbon receptor; AKT, protein kinase B; AMPK, AMP-activated protein kinase; ARE, antioxidant response element; Ca^2+^, calcium ion; DHA, docosahexaenoic acid; EGCG, epigallocatechin-3-gallate; EPA, eicosapentaenoic acid; FFAR2, free fatty acid receptor 2; FFAR3, free fatty acid receptor 3; FFAR4, free fatty acid receptor 4; GPR41, G-protein-coupled receptor 41; GPR43, G-protein-coupled receptor 43; GPR109A, G-protein-coupled receptor 109A; HCAR2, hydroxycarboxylic acid receptor 2; HDACs, histone deacetylases; KEAP1, Kelch-like ECH-associated protein 1; mTORC1, mechanistic target of rapamycin complex 1; NF-κB, nuclear factor kappa B; Nrf2, nuclear factor erythroid 2–related factor 2; PI3K, phosphoinositide 3-kinase; PPARα, peroxisome proliferator-activated receptor alpha; PPARγ, peroxisome proliferator-activated receptor gamma; ROS, reactive oxygen species; SCFAs, short-chain fatty acids; SIRT1, sirtuin 1; TRPV1, transient receptor potential vanilloid 1.

Although phytochemicals and polyunsaturated fatty acids differ structurally, studies show that these molecules converge on a relatively restricted set of intracellular signaling pathways [22,56] (Figure 2). The interaction between these pathways constitutes an integrated system that regulates inflammation, energy metabolism, the antioxidant response, and cell survival.

### 2.1. Modulation of Inflammatory Response: NF-κB Pathway and NLRP3 Inflammasome

Chronic low-grade inflammation represents one of the main pathophysiological mechanisms involved in obesity, type 2 diabetes, cardiovascular diseases, fatty liver disease associated with metabolic dysfunction, inflammatory bowel diseases, and various neoplasms. In this context, nuclear factor κB (NF-κB) plays a central role in regulating the inflammatory response by controlling the expression of genes involved in producing proinflammatory cytokines, chemokines, adhesion molecules, and enzymes such as cyclooxygenase-2 (COX-2) and inducible nitric oxide synthase (iNOS) [66,67]. Several BC can inhibit activation of the NF-κB pathway through various mechanisms. Curcumin, resveratrol, quercetin, epigallocatechin gallate (EGCG), anthocyanins, and capsaicin reduce phosphorylation of the inhibitor of κB (IκB), preventing the nuclear translocation of the NF-κB complex and, consequently, decreasing the expression of inflammatory mediators such as TNF-α, IL-1β, and IL-6. In addition to reducing the systemic inflammatory response, these effects help preserve intestinal barrier integrity, reduce inflammatory cell infiltration, and improve insulin sensitivity in different experimental and clinical models [68,69,70,71].

NLR family pyrin domain containing 3 (NLRP3) inflammasome is a multiprotein complex responsible for activating caspase-1 and the subsequent maturation of the cytokines IL-1β and IL-18. Exacerbated NLRP3 activation has been associated with metabolic inflammation, insulin resistance, atherosclerosis, neuroinflammation, and inflammatory bowel disease. Different BCs, such as flavonoids, terpenes, and phenolic compounds, can attenuate this activation by reducing oxidative stress, modulating mitochondrial function, and inhibiting reactive oxygen species (ROS) production, establishing a functional interaction between the NF-κB, Nrf2, and NLRP3 pathways [72,73].

### 2.2. Modulation of the Nrf2/Keap1 Antioxidant Pathway

Maintaining a balance between reactive oxygen species production and endogenous antioxidant systems is essential for preserving cellular function. The Nuclear factor erythroid 2-related factor 2 (Nrf2) is the main regulator of the antioxidant response, promoting the expression of cytoprotective enzymes, such as heme oxygenase-1 (HO-1), NAD(P)H quinone oxidoreductase 1 (NQO1), glutathione S-transferase, and superoxide dismutase. Under physiological conditions, Nrf2 remains associated with the Keap1 protein in the cytoplasm. However, in the face of oxidative stimuli or the presence of certain BC, their dissociation, nuclear translocation, and activation of genes involved in antioxidant defense occur [56,74,75]. Several phytochemicals, including curcumin, sulforaphane, resveratrol, quercetin, and catechins, act as inducers of the Nrf2 pathway, increasing endogenous antioxidant capacity and reducing oxidative damage to DNA, proteins, and lipids. Beyond direct protection against oxidative stress, Nrf2 activation exerts an indirect anti-inflammatory effect by inhibiting NF-κB-mediated signaling, showing a crosstalk between these two molecular pathways. This interaction explains why many BC have both antioxidant and anti-inflammatory properties, constituting a protective mechanism against metabolic, cardiovascular, and neurodegenerative diseases [14,76,77,78].

### 2.3. Metabolic Sensors: AMPK, mTOR, SIRT1, and PPARs

**Figure 3:**
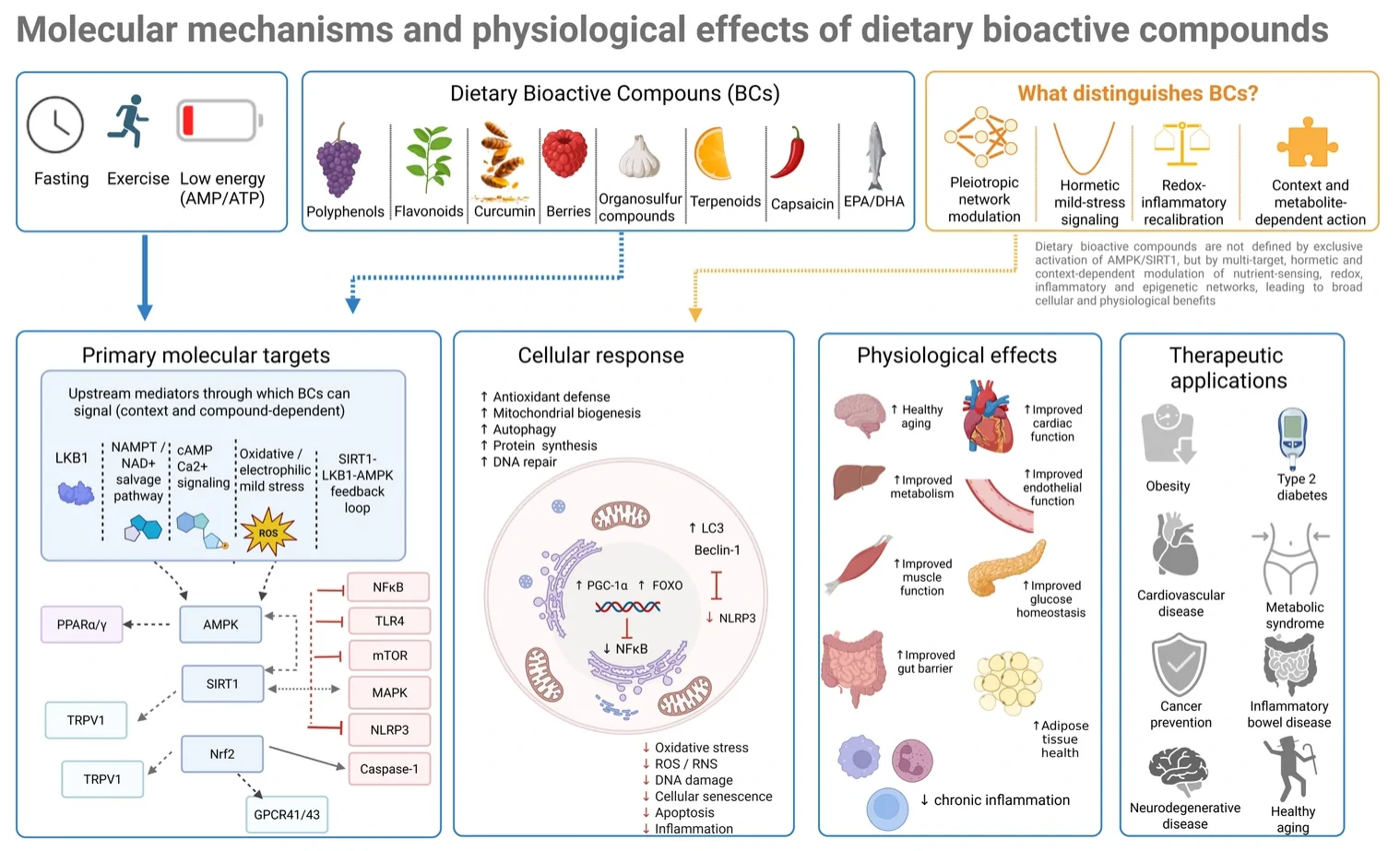
Dietary bioactive compounds modulate nutrient-sensing and stress-response networks through multi-target and context-dependent mechanisms. Conventional metabolic signals, including fasting, exercise, and low-energy status (AMP/ATP), are established regulators of AMPK and SIRT1. Dietary bioactive compounds (BCs) can also modulate AMPK/SIRT1 but are not defined by their exclusive activation; rather, they exert pleiotropic, hormetic, redox-inflammatory, and context-dependent effects across multiple signaling networks. Potential mechanisms linking BCs to AMPK/SIRT1 include LKB1, NAMPT/NAD^+^ salvage, cAMP/Ca^2+^ signaling, mild oxidative/electrophilic stress, and SIRT1–LKB1–AMPK feedback. BCs may simultaneously modulate Nrf2, NF-κB, MAPK, PI3K/AKT, mTOR, TLR4, NLRP3, PPARα/γ, TRPV1, and GPCR41/43, contributing to mitochondrial biogenesis, antioxidant defense, autophagy, redox homeostasis, reduced inflammation, and metabolic adaptation. These mechanisms and effects are dependent on compound, dose, tissue, bioavailability, microbiota-derived metabolites, and cellular context. Solid arrows represent established or broadly supported relationships; dashed arrows represent indirect interactions, signaling crosstalk, or context-dependent relationships. Blue and yellow dotted arrows represent conceptual links between physiological stimuli or dietary BCs and downstream molecular responses. Red T-shaped lines indicate inhibitory effects. Downward and upward arrows indicate reductions and increases, respectively. Bidirectional arrows indicate reciprocal regulatory interactions.. Abbreviations: BCs, bioactive compounds; AMP, adenosine monophosphate; ATP, adenosine triphosphate; AMPK, AMP-activated protein kinase; SIRT1, sirtuin 1; PGC-1α, peroxisome proliferator-activated receptor gamma coactivator 1-alpha; LKB1, liver kinase B1; NAMPT, nicotinamide phosphoribosyltransferase; NAD^+^, nicotinamide adenine dinucleotide; cAMP, cyclic adenosine monophosphate; Ca^2+^, calcium; Nrf2, nuclear factor erythroid 2-related factor 2; NF-κB, nuclear factor kappa B; MAPK, mitogen-activated protein kinase; PI3K/AKT, phosphoinositide 3-kinase/protein kinase B; mTOR, mechanistic target of rapamycin; TLR4, Toll-like receptor 4; NLRP3, NLR family pyrin domain containing 3; PPARα/γ, peroxisome proliferator-activated receptor alpha/gamma; TRPV1, transient receptor potential vanilloid 1; GPR41/GPR43, G protein-coupled receptor 41/43.

In addition to modulating inflammation and oxidative stress, the BC act directly on cellular sensors that control energy metabolism. AMP-activated protein kinase (AMPK) functions as a sensor of cellular energy status, being activated in situations of reduced ATP availability. Its activation promotes fatty acid oxidation, increases glucose uptake, stimulates mitochondrial biogenesis, and inhibits high-energy anabolic pathways, thereby improving insulin sensitivity and metabolic flexibility. Compounds such as resveratrol, catechins, anthocyanins, berberine, and capsaicin demonstrate potential to activate this pathway, mimicking partially metabolic adaptations observed during physical exercise and energy restriction [11,35,79,80,81,82,83] (Figure 3).

mTOR pathway regulates processes related to cell growth, protein synthesis, and proliferation. Although its activation is essential for physiological processes such as tissue growth and regeneration, chronic mTOR hyperactivation is associated with aging, insulin resistance, obesity, and carcinogenesis. Experimental studies suggest that certain BC can indirectly modulate this pathway through AMPK activation and increased SIRT1 activity, favoring autophagy induction and contributing to cellular homeostasis [11,84,85].

SIRT1 is an NAD+-dependent deacetylase involved in regulating energy metabolism, mitochondrial function, and the cellular stress response. Its activation promotes mitochondrial biogenesis, improves insulin sensitivity, and reduces systemic inflammation. Resveratrol represents the most widely studied BC in this context, although other polyphenols can also modulate this pathway [11,84,85].

Peroxisome proliferator-activated receptors (PPARs) constitute another family of molecular targets often modulated by BC. These nuclear receptors regulate genes involved in lipid metabolism, fatty acid oxidation, adipocyte differentiation, and the inflammatory response. PPAR-α activation favors lipid oxidation, while PPAR-γ modulation influences glycemic homeostasis, insulin sensitivity, and anti-inflammatory adipokine production. Different flavonoids, polyunsaturated fatty acids, and other phytochemicals can modulate these receptors, contributing to the metabolic effects observed in experimental and clinical studies [82,86,87,88].

Taken together, this evidence suggests that BC exerts its biological effects through the integrated modulation of interconnected molecular networks, rather than by isolated actions on a single cellular target. This systemic view represents a shift in understanding the relationship between food and health, providing mechanistic bases for explaining the benefits observed in different plant-derived dietary patterns and sustaining the therapeutic potential of these compounds in preventing and managing certain human diseases.

## 3. Bioactive Compounds, Gut Microbiota, and Host Homeostasis

The gut microbiota constitutes a complex ecosystem inhabited by trillions of microorganisms that perform essential functions in nutrient digestion, metabolite production, immune system development, and maintaining intestinal barrier integrity [25].

Advances in sequencing technologies and omics sciences have transformed the understanding of the role of microbiota in human health, demonstrating that its composition and metabolic activity are strongly influenced by diet. In this context, BC emerge as modulators of communication between food, microbiota, and host [89,90,91].

Because of limited absorption, a significant fraction of BC reaches the colon, where it is modified by intestinal microbiota. This process yields metabolites such as low-molecular-weight phenolic acids, urolithins, flavonoid derivatives, and other compounds with high bioavailability and biological activity. In this way, the microbiota amplifies and modifies the physiological effects of BC [92,93]. Moreover, polyphenol-rich diets promote the growth of beneficial bacteria, including Bifidobacterium and Lactobacillus species, as well as butyrate-producing microorganisms such as *Faecalibacterium prausnitzii*, *Roseburia* spp., and *Akkermansia muciniphila*, which are associated with intestinal mucus layer preservation, improved insulin sensitivity, and reduced metabolic inflammation [94]. At the same time, potentially pathogenic microorganisms and pro-inflammatory metabolite production decrease, favoring the reestablishment of intestinal eubiosis [95,96,97].

Changes in microbiota composition directly affect microbial metabolite production, especially short-chain fatty acids (SCFAs). These fatty acids serve multiple physiological functions, providing the main energy source for colonocytes, strengthening tight junctions, stimulating mucin production, and maintaining intestinal barrier integrity. In addition, SCFAs modulate the immune response by activating G-protein-coupled receptors (GPR41, GPR43, and GPR109A) and inhibiting histone deacetylases, mechanisms associated with increased regulatory T cells, reduced pro-inflammatory cytokine production, and maintained intestinal immune tolerance [98,99].

The literature indicates that isolated bioactive compounds and bioactive fractions can preserve intestinal permeability by strengthening tight junctions, stimulating mucin production, and modulating inflammation, oxidative stress, and the microbiota, although most evidence remains preclinical. Among experimental findings, proanthocyanidins normalized LPS-induced permeability in rats [100], while citrus flavonoids and other polyphenols were associated with the protection of tight junctions, the mucus layer, and immune homeostasis [101]. Furthermore, glutamine, curcumin, and bioactive fish peptides reduced colonic hyperpermeability in a murine stress model, demonstrating a combined effect superior to isolated use [102]. Garcinol and compounds from *Garcinia morella* reduced permeability following infection in Caco-2 cells [103], whereas taurine blocked tight junction disruption and putrescine-induced inflammatory exacerbation in mice [104]. Concurrently, butyrate reduced LPS-induced barrier damage in a dose-dependent manner and upregulated claudin-3 and claudin-4 [105].

Resveratrol showed one of the strongest signals in animal models, reducing the lactulose/mannitol ratio by 70.5% at 10 mg/kg and 86.8% at 20 mg/kg, in addition to attenuating tight junction protein disruption [106]. The same systematic review reported benefits for grape seed extract, curcumin, genistein, chlorogenic acid, grape pomace, olive leaf, and cranberry, associated with reduced inflammatory molecules and increased antioxidant enzymes [106].

In humans, the most direct evidence stems from bioactive-rich dietary patterns rather than purified molecules: in 66 older adults with increased permeability, an 8-week polyphenol-rich diet reduced serum zonulin and increased fiber-fermenting, butyrate-producing bacteria [107]. These findings support the clinical plausibility of polyphenols but do not definitively prove the efficacy of a specific isolated compound [98,108].

Nonetheless, individual responses to BC vary widely. Factors such as the composition of basal gut microbiota, genetic background, age, dietary habits, prior medication use, and existing medical conditions play a decisive role in modulating the biotransformation of these molecules and the generation of biologically active metabolites. As a consequence, individuals undergoing the same dietary intervention may respond differently, a factor that has driven the development of precision nutrition and personalized approaches that integrate metabolic profile, gut microbiota, and host genetic characteristics [48,109].

In summary, the interaction between BC and the gut microbiota is a key factor in the benefits attributed to dietary patterns rich in vegetables. Beyond serving as a simple metabolic substrate, the microbiota actively participates in the biotransformation of BC, enhancing or modifying their biological activity, while itself being modulated by these molecules. This bidirectional communication strengthens the concept of the diet–microbiota–host axis as a central element in maintaining homeostasis and preventing chronic diseases [9,110].

## 4. Therapeutic Potential of Bioactive Compounds in Human Diseases

The growing understanding of the molecular mechanisms modulated by BC has increased interest in their use to prevent and treat NCDs [7,111].

Unlike pharmacological approaches, which are conventionally directed at a single molecular target, the pleiotropic effects of BC on inflammation, oxidative stress, energy metabolism, intestinal barrier integrity, and immune response make it a potential candidate for reducing the impact of these diseases [23,35,112,113,114,115].

Although these characteristics suggest that the same class of compounds may be effective in different clinical conditions that share similar pathophysiology (such as chronic inflammation, insulin resistance, mitochondrial dysfunction, and dysbiosis), clinical studies remain limited [116,117].

It should be noted that AMPK and SIRT1 are not exclusive targets of bioactive compounds: they are also activated by conventional physiological and nutritional stimuli, such as caloric restriction, physical exercise, and fasting-associated changes in the cellular AMP/ATP and NAD^+^/NADH ratios. What distinguishes dietary bioactive compounds in this context is less a unique signaling mechanism than their capacity to pharmacologically mimic or potentiate these energy-sensing pathways independently of caloric restriction—for instance, through direct binding to sirtuin activators or AMPK-upstream kinases—and to simultaneously modulate redox and inflammatory targets (Nrf2, NF-κB) alongside AMPK/SIRT1. Whether compound-specific, receptor-level signaling mechanisms exist for each BC class, as opposed to convergent downstream effects on shared energy-sensing nodes, remains an open question that warrants further mechanistic investigation.

### 4.1. Obesity, Metabolic Syndrome, and Type 2 Diabetes

Obesity and type 2 diabetes are characterized by persistent metabolic inflammation, associated with insulin resistance, increased production of reactive oxygen species (ROS), and changes in gut microbiota composition [7,118,119,120,121]. In preclinical studies, some BC demonstrate potential to modulate these processes by activating AMPK, inhibiting the NF-κB and NLRP3 pathways, and regulating PPAR receptors. Polyphenols such as resveratrol, catechins, anthocyanins, and quercetin, as well as compounds such as curcumin and capsaicin, have been linked to increased fatty acid oxidation, improved peripheral glucose uptake, reduced hepatic lipogenesis, and decreased production of pro-inflammatory cytokines [99,122,123,124]. At the same time, experimental models demonstrated the capacity of these compounds to modulate the gut microbiota, promoting increased production of short-chain fatty acids (especially butyrate and acetate), in addition to improving the integrity of the intestinal barrier and reducing metabolic endotoxemia, with favorable repercussions on insulin sensitivity and glycemic control [98,125,126,127].

The literature on BC and obesity over the past 10 years includes human clinical trials evaluating green tea catechins, resveratrol, berberine, curcumin, anthocyanins, hydroxycitric acid, alpha-lipoic acid, and certain probiotics. In contrast, numerous other molecules remain predominantly in the preclinical phase [128,129,130]. The core issue is not a paucity of studies, but rather that clinical outcomes are more consistent for specific compounds and remain limited by heterogeneity, bioavailability, and a lack of standardization [128,129,131].

The primary limitation is translational: many isolated compounds continue to be supported mainly by cellular and animal models rather than robust human clinical trials [126,132,133]. This applies to candidates such as nanoformulated quercetin, *Annona squamosa* fractions, *Kalanchoe pinnata*, *Dendrobium* metabolites, and millet-derived compounds, which currently offer primarily mechanistic or preclinical evidence [132,134,135,136,137].

In recent years, reviews focusing on human trials with polyphenols have summarized data on resveratrol, quercetin, curcumin, and related extracts; however, they highlighted contrasting results across studies due to variations in study design, duration, and chemical formulation [131]. In parallel, reviews on anthocyanins concluded that foods and compounds rich in this class had already been evaluated in clinical studies, showing benefits primarily on obesity-associated inflammation [138].

More recent reviews maintain that clinical evidence is becoming more robust for a restricted group of molecules. EGCG, resveratrol, berberine, anthocyanins, curcumin, hydroxycitric acid, and α-lipoic acid have the strongest clinical support; however, there are still few clinical trials, variable outcomes, and incomplete adverse event data [128].

Another recent review attributed promising clinical outcomes to green tea extract, *Garcinia cambogia*, resveratrol, and berberine, showing effects on weight, BMI, and lipid profile. However, it still calls for more rigorous clinical validation [129]. Consistently, recent reviews reported measurable fat reductions of 4% to 5% in clinical trials utilizing resveratrol at 500 mg/day and green tea catechins at 300–400 mg/day; additionally, berberine at 500 mg three times daily demonstrated significant weight loss, and curcumin at 1 g/day showed effects on appetite via the gut–brain axis [139].

The strongest evidence pertains to catechins, resveratrol, berberine, and curcumin. Catechins and resveratrol demonstrated a 4–5% reduction in body fat, accompanied by metabolic improvements, in human trials [139]. Berberine consistently ranks among the compounds with the strongest clinical support, demonstrating metabolic improvements and weight loss in human studies [129,139]. Curcumin has more moderate clinical evidence, with signs of efficacy for metabolism and appetite regulation that remain dependent on formulation and bioavailability [129,139]. Among probiotics, some randomized trials reported reductions in visceral fat, waist circumference, and insulin resistance, but outcomes vary by strain and intervention duration [140].

The literature points out three major limitations: low bioavailability, lack of standardized dosing, and variability across formulations and patient cohorts [128,139]. Consequently, the implication is clear: while clinical studies on isolated bioactive compounds in obesity already exist, broader clinical application still depends on standardized, larger, long-term trials [141,142,143,144,145].

Type 2 Diabetes (T2DM): Research on isolated bioactive compounds for T2DM management has progressed steadily, with an emphasis on berberine, resveratrol, curcumin, α-lipoic acid, and specific micronutrients. Most of the available scientific literature remains highly heterogeneous, often confounding the evaluation of crude extracts, functional foods, and multi-ingredient supplements. Consequently, few isolated compounds possess a robust, reproducible, and standardized clinical evidence base [146,147,148].

Although comprehensive systematic reviews and meta-analyses indicate consistent improvements in glycemic control and reduced oxidative stress, validation of these compounds as reliable adjuvant therapies remains limited. A persistent shortage of large-scale, independently replicated RCTs using standardized dosages and formulations remains [149].

The strength of clinical evidence varies considerably among the isolated bioactives investigated. The most consolidated clinical support currently focuses on alpha-lipoic acid and vitamin D supplementation. A robust meta-analysis encompassing 109 RCTs in patients with T2DM consistently demonstrated a reduction in glycated hemoglobin (HbA1c). Additionally, curcumin reduced malondialdehyde levels, a critical biomarker of systemic oxidative stress [149].

Other polyphenols and alkaloids show relevant therapeutic potential, though clinical data remain preliminary. Resveratrol has shown promising antidiabetic activity in preliminary clinical trials, improving glycemic control and glucose homeostasis in individuals with insulin resistance [147,150,151]. Berberine also presents a promising body of clinical evidence for T2DM. Recent advances with optimized derivatives, such as berberine ursodeoxycholate, suggest an efficacy comparable to metformin in metabolic control, with the additional benefit of a lower incidence of gastrointestinal adverse effects. Despite this potential, validating this alternative requires confirmation in larger clinical cohorts [152].

Cesar et al. [153] conducted a 26-week, double-masked, randomized, placebo-controlled, crossover trial in individuals with prediabetes, evaluating a citrus flavonoid supplement (administered at 250 mg/day) as an adjuvant to metformin. The authors reported a 5% reduction in 2 h oral glucose tolerance test (OGTT) values, preservation of active GLP-1, a 12% reduction in TNF-α, and a 7.5% increase in ferric reducing antioxidant power (FRAP), alongside minor reductions in body weight, body fat, BMI, and systolic blood pressure.

**Figure 4:**
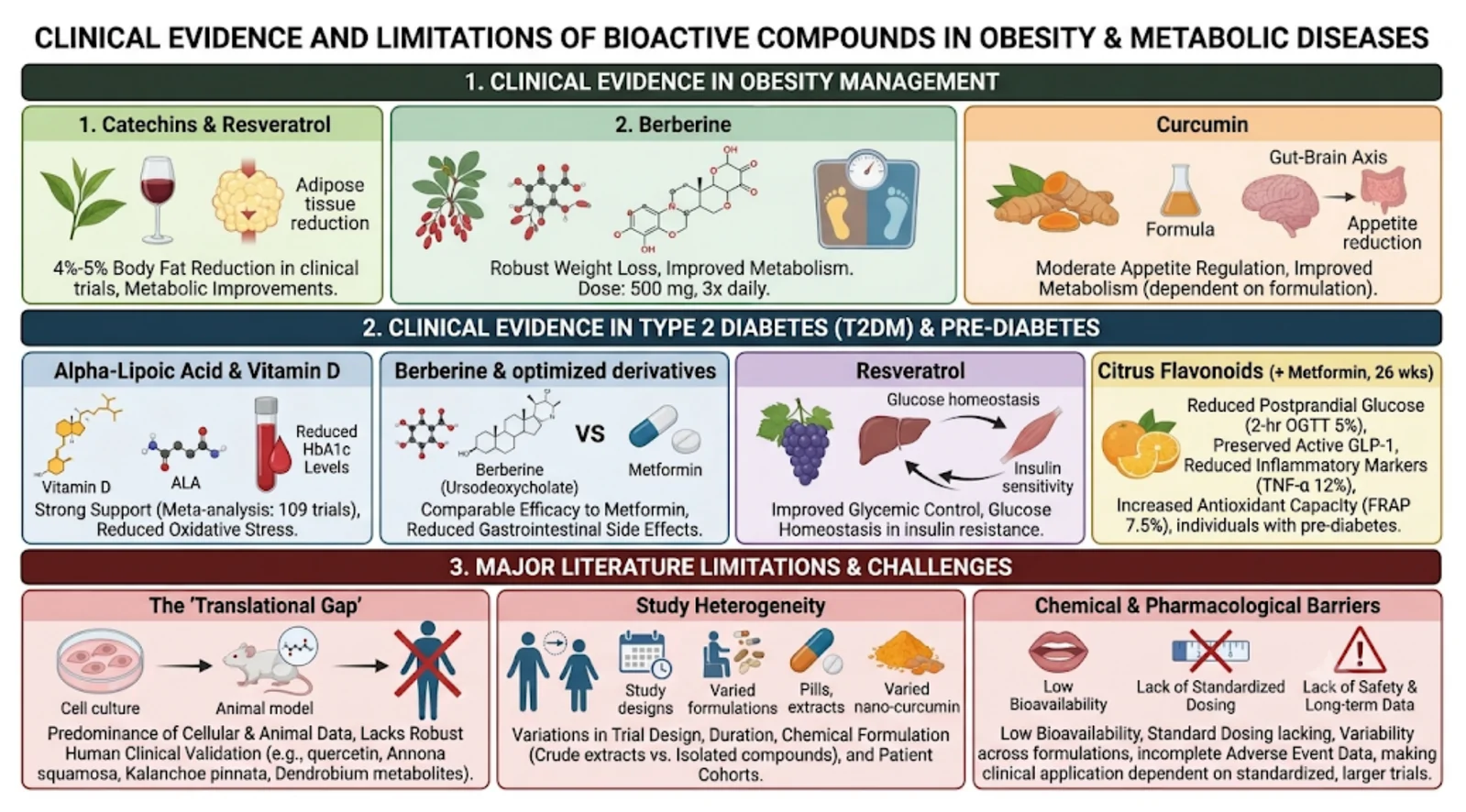
Scientific summary of clinical evidence and literature limitations for bioactive compounds in obesity and type 2 diabetes management. Green tea catechins and resveratrol show a 4–5% reduction in body fat, berberine shows robust support for weight loss (500 mg, three times daily), and curcumin shows moderate efficacy in appetite regulation via the gut–brain axis. For type 2 diabetes and pre-diabetes, alpha-lipoic acid and vitamin D supplementation provide the strongest evidence for reducing glycated hemoglobin (${\mathrm{HbA}}_{1c}$), with additional promising results for optimized berberine derivatives, resveratrol, and citrus flavonoids combined with metformin. Major literature limitations include translational gaps, study heterogeneity, and pharmacological barriers such as low bioavailability and lack of standardized dosing, highlighting the need for larger, long-term clinical trials. Image created by the author using This Figure was created, edited and refined using Google Gemini (Imagen 3 model) (2026).

As illustrated in Figure 4, a substantial translational gap persists between the robust preclinical and early-phase clinical signals summarized above and their confirmation in adequately powered, long-term human trials. This gap arises from several compounding factors: preclinical models typically employ compound concentrations several-fold higher than those achievable through oral human dosing; interspecies differences in absorption, first-pass hepatic metabolism, and gut-microbial biotransformation alter the identity and potency of the circulating bioactive metabolites; and clinical heterogeneity in dose, formulation, treatment duration, and patient phenotype further limits the comparability of human findings across studies. Together, these factors make it difficult to establish whether the metabolic benefits observed for compounds such as berberine, catechins, or citrus flavonoids reflect a true, clinically meaningful effect size or are partly an artifact of small, short-duration trials; this uncertainty is a central reason why none of the bioactive compounds discussed here can yet be recommended as a standardized adjuvant therapy for obesity or type 2 diabetes.

### 4.2. Cardiovascular Diseases

Vascular inflammation and oxidative stress play a central role in the pathophysiology of cardiovascular diseases. In this context, several BC exert cardioprotective effects through complementary mechanisms, including reducing low-density lipoprotein (LDL) oxidation, improving endothelial function, decreasing platelet aggregation, and modulating lipid metabolism [154,155,156] (Table 1).

**Table 1:** Clinical evidence on dietary bioactive compounds for cardiovascular health *.

Compound	Clinical Condition	RCT Evidence Strength	Dose	Duration	N Participants	Outcome Type	Key Clinical Findings
Alpha-lipoic acid (ALA)	Acute ischemic stroke [157]	Low	1200 mg/day	3 weeks	40 completed; 42 randomized.	Inflammatory, oxidative, clinical prognosis	Within-group decreases in TNF-α and IL-6, but no significant difference versus control.
Alpha-lipoic acid (ALA)	PCOS [158]	Moderate	600–1800 mg/day	Median 15 weeks, range 6–25	7 RCTs; studies with 38–71 women	Glycemic, hormonal, lipid, oxidative, anthropometric	Improved FBS and HOMA-IR, without consistent improvement in lipids, hormones, or BMI
Alpha-lipoic acid (ALA)	MetS and related disorders [159]	Moderate	Subgroup analyses used >600 vs. ≤600 mg/day.	Subgroups <8 vs. ≥8 weeks	18 RCTs	Inflammatory	Reduced CRP, IL-6, and TNF-α, but with high heterogeneity
Anthocyanins	T2DM in general [160]	Moderate	Variable doses and formulations across studies	Generally short duration	18 recent clinical studies; 14 RCTs in one synthesis	Glycemic, inflammatory, vascular	Human literature shows improved diabetes-related outcomes, especially in at-risk groups, but with limitations in duration, sample size, and heterogeneity.
Anthocyanins	T2DM, at-risk group, and healthy individuals [161]	Moderate	320 mg/day	4 weeks	40 participants	Inflammatory and glycemic	Reduced IL-6, IL-18, and TNF-α in T2DM; improved FBG, LDL, and uric acid in the at-risk group
Anthocyanins/blueberries	Metabolic syndrome [162]	Moderate to high	75 or 150 g/day	6 months	115 participants	Vascular function	150 g/day improved FMD and AIx; 75 g/day had no effect
Berberine	Metabolic syndrome [163]	Moderate to high	300–1500 mg/day	84–140 days	12 RCTs; 889 patients	Glycemic, lipid, anthropometric, blood pressure, safety	Reduced TG, FPG, waist circumference, LDL, TC, and 2hOGTT, but not HDL-C, SBP, or DBP
Berberine	T2DM/prediabetes [164]	High	Variable across studies; interventions with berberine or Rhizoma Coptidis	4 weeks to 6 months	4158 participants; 46 trials	Glycemic, insulin resistance, lipid, inflammatory, safety	Reduced HbA1c, FPG, 2hPG, HOMA-IR, BMI, TG, TC, and LDL; strong evidence as adjuvant therapy
Catechins/EGCG	Postmenopausal dyslipidemia [165]	Moderate	1315 mg catechins/day, 843 mg EGCG	12 months	538 GTE, 537 placebo; 936 completed substudy	Lipid	Reduced TC, LDL, and non-HDL; HDL unchanged and TG increased slightly
Catechins/green tea extract	MetS and healthy adults [166]	Moderate	890 mg/day total catechins	4 weeks	40 total participants	Glycemic, gut, permeability, systemic inflammatory, lipid Glycemic, gut, lipid	Reduced endotoxin, calprotectin, myeloperoxidase, and fasting glucose, with no effect on lipids, BP, TNF-α, or IL-6
Catechins + inulin	Visceral obesity [167]	Moderate for HOMA-IR; low for visceral fat	400 mg/day catechins + 2.3 g/day inulin	12 weeks	96 randomized	Insulin resistance, visceral adiposity, microbiota	Improved HOMA-IR, but did not reduce visceral fat
Curcumin	MetS/related conditions [168]	High	80–4000 mg/day, with wide heterogeneity	Generally 8–12 weeks; few studies >6 months	104 RCTs	Inflammatory, glycemic, lipid, oxidative	More consistent benefits on glycemia and inflammation; oxidative markers less consistent
Curcumin	T2DM [168]	High	80–4000 mg/day; most common 500–1000 mg/day; nanoformulations effective at 80 mg/day	Predominantly 8–12 weeks	104 RCTs in the pooled set	Glycemic, inflammatory, lipid, oxidative	Reduced FBG, HbA1c, TG, LDL, CRP, TNF-α, and IL-6; effect depends on formulation
Ferulic acid {Citation}	Hyperlipidemia	Moderate	1000 mg/day (Bumrungpert et al., 2018 [162])	6 weeks (Bumrungpert et al., 2018 [162])	48 participants (Bumrungpert et al., 2018 [162])	Lipid, inflammatory, oxidative	Reduced TC, LDL-C, TG, hs-CRP, TNF-α, MDA, and ox-LDL; small study with surrogate outcomes (Bumrungpert et al., 2018 [162])
Palm phenolics (OPP)	Healthy adults [162]	Low to moderate	250, 1000, or 1500 mg/day	60 days	97 participants	Blood pressure and antioxidant	250 mg/day reduced SBP/DBP; 1500 mg/day increased total antioxidant capacity
Purified anthocyanins	Prediabetes/early diabetes [162]	Moderate for null CV effect	160, 320, or 640 mg/day	12 weeks	184 participants	Cardiovascular surrogate	No change in arterial stiffness or cardiovascular risk markers
Resveratrol	T2DM [169,170]	Moderate	40–1000 mg/day [170] 200 mg/day [169]	4–24 weeks [170] 24 weeks [169]	6 RCTs; 533 participants [170] 110 randomized; 94 completed [169]	Inflammatory and oxidative Glycemic, inflammatory, oxidative	Reduced CRP, lipid peroxides, 8-isoprostanes, and oxidative stress score; low or very low certainty of evidence [170] Reduced glucose, insulin, HOMA-IR, MDA, hs-CRP, TNF-α, and IL-6 [169]

* Data extraction was assisted by the Consensus artificial intelligence platform. The AI-generated initial search and synthesis were subsequently verified against the original peer-reviewed sources. All extracted data, clinical outcomes, and limitations were independently critically analyzed, validated, and finalized by the authors, who take full responsibility for the accuracy and integrity of the content.

Flavonoids present in berries, cocoa, and green tea, as well as omega-3 fatty acids found in marine fish, show consistent evidence of reduced inflammatory biomarkers and improved vascular function [171,172,173,174].

These effects are mainly related to the activation of the Nrf2 pathway, increased bioavailability of nitric oxide, inhibition of NF-κB, and modulation of PPAR receptors, contributing to attenuation of vascular inflammation and progression of atherosclerosis [175,176,177,178,179].

In humans, isolated bioactive compounds appear to improve cardiovascular risk factors, particularly lipids, blood pressure, and endothelial function; however, evidence for reductions in myocardial infarction, stroke, or mortality remains insufficient [180,181,182]. Furthermore, these effects vary by compound, dose, duration, and participants’ baseline profile, with more pronounced responses generally observed in individuals at higher cardiometabolic risk [181,183].

Meta-analyses of clinical trials demonstrate that phytosterols reduce total cholesterol, LDL, triglycerides, and blood pressure, although most studies are short-term [182]. Flavonols also improve multiple biomarkers, reducing total cholesterol, LDL, triglycerides, fasting blood glucose, and blood pressure; however, the overall quality of evidence was moderate-to-low for several outcomes [181].

Regarding endothelial function, chlorogenic acids (CGAs) increased flow-mediated dilation in randomized trials, with a stronger effect observed for CGA-rich beverages compared to isolated supplements. In contrast, caffeine showed no benefit and hydroxyhydroquinone (HHQ) reduced the vascular response [180]. Broader reviews on polyphenols conclude that while certain compounds improve specific markers, long-term human outcomes remain inconsistent and do not yet support definitive recommendations [184].

A randomized, double-masked, placebo-controlled clinical trial with 99 participants aged 60–80 years, evaluating 320 mg/day of anthocyanins for 24 weeks, showed that this supplementation reduced C-reactive protein (CRP) levels, particularly in participants with elevated baseline inflammation. Anthocyanin intake was also associated with changes in low-density lipoprotein (LDL) cholesterol, cardiometabolic scores, interleukin-6 (IL-6), and interleukin-1β (IL-1β) [185].

Regarding the effect of anthocyanin on CRP, a systematic review and meta-analysis evaluated the effects of purified anthocyanins on circulating C-reactive protein (CRP) levels [186]. The analysis demonstrated that anthocyanin supplementation did not reduce CRP. Furthermore, subgroup analyses stratified by intervention duration, dosage, participant health status, or the specific form of anthocyanin administered (purified versus extract) yielded consistently non-significant results.

However, a systematic review and meta-analysis of 32 randomized clinical trials demonstrated that dietary anthocyanins significantly attenuate systemic and vascular inflammation [172]. The pooled analysis revealed substantial reductions in key inflammatory biomarkers, including CRP by 0.33 mg/L, interleukin-6 (IL-6) by 0.41 pg/mL, and tumor necrosis factor-alpha (TNF-α) by 0.64 g/mL, whereas no significant alterations were observed for IL-1β or P-selectin. Notably, subgroup analyses indicated a dose-dependent efficacy, wherein daily doses exceeding 300 mg not only induced more pronounced decreases in these primary cytokines but also significantly down-regulated vascular cell adhesion molecule-1 (VCAM-1).

Taken together, these apparently contradictory findings on anthocyanins and CRP likely reflect differences in dose (with benefit concentrated above ~300 mg/day), anthocyanin form (purified compound versus whole-food extract), intervention duration, and the baseline inflammatory status of the population studied, rather than a true absence of anti-inflammatory activity; this pattern illustrates why compound-level meta-analyses that pool heterogeneous dosing and population strata may underestimate effects that are, in fact, dose- and population-dependent.

Clinical trials evaluating resveratrol in high-risk populations have demonstrated favorable effects of isolated resveratrol on cardiovascular surrogate outcomes. In patients with chronic kidney disease and diabetes, administration of 400 mg/day for 6 weeks increased flow-mediated dilation by 43% compared to placebo; however, this intervention did not alter blood pressure, glycated hemoglobin (HbA1c), or estimated glomerular filtration rate (eGFR) [187].

In hypertensive subjects, long-term treatment (400 mg/day for 6 months) reduced left atrial remodeling and decreased circulating fibrosis biomarkers. These changes were not associated with significant modifications in left ventricular structure [188]. Additionally, in patients with heart failure and reduced ejection fraction, 100 mg/day for 3 months improved systolic and diastolic function, exercise capacity, and quality of life, while reducing prognostic markers such as NT-proBNP and galectin-3. However, this finding is limited by the small sample size and the protocol’s short follow-up period [189].

In patients with type 2 diabetes, interventions using 1000 mg/day for 8 weeks demonstrated specific benefits, including a reduction in fasting plasma glucose (Δ=−7.97±13.6 mg/dL), decreased insulin levels, and increased HDL-cholesterol, with no changes in anthropometric measurements [190]. Similarly, a 24-month crossover study in postmenopausal women showed that a split dose of 75 mg twice daily improved cerebrovascular responsiveness, fasting insulin, and insulin resistance indices [191].

Conversely, many clinical trials failed to replicate these metabolic benefits. Targeted studies evaluating the compound’s impact on composite cardiovascular indices in type 2 diabetes, cardiometabolic risk in overweight individuals with insulin resistance, and glucose levels and cardiovascular risk factors in schizophrenia yielded mostly neutral results [192,193].

Bumrungpert et al. (2018) [162] in a randomized, placebo-controlled clinical trial (Level 1b evidence), demonstrated that isolated ferulic acid supplementation reduced inflammatory markers and oxidative stress. However, the limited sample size (n = 48) and reliance on surrogate endpoints limit the evidence to moderate quality, indicating that larger longitudinal cohorts are needed to confirm reductions in hard cardiovascular outcomes.

The dose–response study by Liu et al. [194] (Level 1b) showed that 12 weeks of purified anthocyanin supplementation failed to attenuate arterial stiffness in hyperglycemic older adults. This finding suggests that chronic vascular damage (such as that mediated by advanced glycation end-products or AGEs) is not easily reversed by an isolated antioxidant, reinforcing the premise that phytochemical efficacy relies on the collaboration of a complex food matrix rather than isolated compounds.

Addressing pharmacokinetic barriers, Fairus et al. [195] conducted a Phase 1 trial (Level 2b/3) demonstrating that encapsulated oil palm phenolics improved systemic antioxidant status and blood pressure. Although evidence from healthy cohorts remains low quality for clinical guideline formulation regarding pathology management, this trial provides a pharmacodynamic proof of concept for delivery bioengineering.

Curtis et al. (2019) [196] (Level 1b, High GRADE Quality) evidenced that daily blueberry consumption for 6 months in metabolic syndrome patients improved endothelial function and cardiometabolic biomarkers. This study highlights the therapeutic superiority of whole-food matrices over isolated compounds.

The STRENGTH megatrial [197] evaluated high-dose EPA and DHA versus corn oil in over 13,000 high-risk patients. The trial found no reduction in major adverse cardiovascular events (MACE). It raised critical safety concerns regarding an increased risk of incident atrial fibrillation, discouraging the universal prescription of high-dose marine omega-3 for hard clinical outcomes. However, subsequently published meta-analyses concluded that omega-3 reduces cardiovascular mortality, non-fatal myocardial infarction, coronary events, major adverse cardiovascular events (MACE), and the need for revascularization; it also improves endothelial function as measured by flow-mediated dilation (FMD) [198,199].

### 4.3. Inflammatory Bowel Diseases

Inflammatory bowel diseases (IBD), such as ulcerative colitis and Crohn’s disease, arise from a complex interplay of genetic susceptibility, environmental exposures, dysbiosis, and aberrant mucosal immune responses [71,200,201]. Chronic barrier dysfunction and microbial imbalances sustain this inflammatory state through persistent activation of key cascades—including NF-κB, NLRP3, MAPK, TLR4/MyD88, and JAK/STAT signaling—resulting in overproduction of pro-inflammatory cytokines such as TNF, IL-1β, IL-6, and IL-8 [202,203,204,205,206].

In this context, dietary- and plant-derived bioactive compounds (including curcumin, quercetin, resveratrol, catechins, sulforaphane, berberine, luteolin, genistein, anthocyanins, proanthocyanidins, polysaccharides, and ω-3 polyunsaturated fatty acids) have emerged as promising adjunctive strategies due to their ability to simultaneously target inflammation, oxidative stress, epithelial permeability, and dysbiosis [207,208,209,210,211].

These compounds include not only curcumin, quercetin, resveratrol, and catechins, but also sulforaphane, berberine, luteolin, genistein, anthocyanins, proanthocyanidins, polysaccharides, ω-3 polyunsaturated fatty acids, and other plant-derived constituents that converge on anti-inflammatory, antioxidant, barrier-protective, and microbiota-modulating functions [212,213,214,215].

On a molecular level, these compounds attenuate mucosal inflammation by suppressing NF-κB, JAK/STAT, and MAPK signaling, thereby dampening immune cell activation and cytokine production [205,215].

These anti-inflammatory and antioxidant actions are reinforced by barrier-oriented mechanisms; by enhancing tight junction stability and mucin production, bioactives directly limit paracellular antigen translocation and break the cycle of chronic inflammation [201,216]. This therapeutic efficacy is closely tied to the host–microbiota–metabolite axis. Active IBD is characteristically marked by a depletion of fiber-fermenting, short-chain fatty acid (SCFA)-producing bacteria, leading to diminished levels of acetate, propionate, and butyrate [201,206].

This decline impairs epithelial energy supply, compromises barrier integrity, and disrupts tolerogenic signaling through G-protein coupled receptors like GPR41, GPR43, and GPR109A [201,217].

Bioactive compounds help restore these vital metabolic networks by reshaping the microbiota and stimulating the production of SCFAs, bile acid derivatives, and tryptophan metabolites [110,218]. For instance, curcumin administration in colitis models correlates with increased *Akkermansia* abundance and elevated levels of butyrate, propionate, and tryptophan pathways, illustrating a dual host–microbiome metabolic modulation [219].

Despite this potential, the clinical signal remains strongest for curcumin, particularly in maintaining ulcerative colitis remission while most other bioactives are still supported predominantly by preclinical data [71,200,211]. Consequently, current evidence positions these compounds as adjunctive rather than stand-alone therapies [71,200,220]. Major translational bottlenecks persist, including low bioavailability, colonic instability, inconsistent dosing, interindividual microbiota variability, and potential drug interactions [71,200,220].

To overcome these limitations, current research focuses on advanced strategy integration, such as nanoparticle delivery systems, polysaccharide-based encapsulation, multi-target compound discovery, and multi-omics profiling to improve targeted colonic delivery and patient stratification [216,219,221].

### 4.4. Cancer and Neurodegenerative Diseases

Oxidative stress, low-grade chronic inflammation, and mitochondrial dysfunction are also intimately involved in the pathophysiology of several neoplasms and neurodegenerative diseases [222,223].

Experimental evidence demonstrates that BC can modulate processes related to cell proliferation, apoptosis, autophagy, and genomic stability by regulating the Nrf2, NF-κB, SIRT1, and mTOR pathways. Compounds such as resveratrol, curcumin, sulforaphane, and epigallocatechin gallate have the potential to reduce neuroinflammation, preserve mitochondrial function, and limit out-of-control proliferative processes in experimental models [224,225,226,227,228].

Despite the solid mechanistic foundation in vitro and in vivo, the original studies in humans and the translational approaches are still markedly heterogeneous between different compounds, formulations, and stages of disease, especially not yet targeted at neurodegeneration [229]. Thus, these compounds cannot yet be recommended as specific therapies or substitutes for cancer or neurodegenerative diseases [230,231].

The most consistent potential of these molecules resides in the field of chemoprevention and as a complementary or adjuvant strategy, whose clinical success still depends on overcoming limitations in pharmacokinetics, bioavailability, and standardization of formulations [232,233,234,235].

Taken together, the available evidence demonstrates that BC have the potential to act in different pathological spectra through integrated modulation of inflammatory, oxidative, metabolic, and immunological pathways [236,237] (Table 2).

**Table 2:** Clinical evidence for dietary bioactive compounds in metabolic, cardiovascular, intestinal, and neurodegenerative diseases *.

Clinical Condition	Compounds with Strongest Evidence	Main Clinical Effects	Type of Study	Number of Participants/Studies	Main Limitations	Strength of Evidence
Obesity/Type 2 diabetes	Curcumin, resveratrol, anthocyanins, capsaicin [238,239,240,241,242]	Curcumin reduces fasting glucose, HbA1c, triglycerides, LDL, and systemic inflammation; anthocyanins show modest reductions in HbA1c and cytokines; resveratrol has mixed results.	RCTs aggregated in systematic reviews and meta-analyses, with individual long-term RCTs in T2D.	104 RCTs in the Curcuma longa meta-analysis; 105 studies in the comparative review; individual RCTs with 229 randomized and 114 participants.	Moderate to high heterogeneity among formulations, dose, and duration; many small studies; low bioavailability; inconsistent and generally modest resveratrol effects.	Moderate to high.
Cardiovascular disease	Flavonoids, omega-3 fatty acids [198,199]	Omega-3 reduces CV mortality, non-fatal MI, coronary events, MACE, and revascularization; improves endothelial function measured by FMD.	Meta-analyses of RCTs with clinical and vascular outcomes.	38 RCTs/149,051 participants for CV outcomes; 16 RCTs/901 participants for FMD; 13 studies/853 participants in chronic heart disease.	Increased risk of atrial fibrillation, especially with EPA; potential increased bleeding; clinical heterogeneity among trials,	High.
Inflammatory bowel disease	Curcumin, quercetin, catechins, sulforaphane, ω-3 PUFA, polysaccharides [220,243,244,245,246,247]	Curcumin improves remission and clinical response in ulcerative colitis; polyphenols elevate SCFA, reduce inflammatory cytokines, suppress NF-κB, and protect the intestinal barrier.	Placebo-controlled RCTs of curcumin, supported by translational and mechanistic reviews.	13 RCTs in the clinical meta-analysis; 322 UC + 308 placebo and 51 CD + 41 placebo; broad review with 67 studies.	More consistent benefit in UC than in Crohn’s; heterogeneity in dose, duration, and formulation; a significant portion of the base remains pre-clinical; bioavailability remains a limiting factor.	Low to moderate.
Cancer/Neurodegeneration	Resveratrol, sulforaphane, EGCG [242,248,249]	Human evidence shows improvement mainly in biomarkers of oxidative stress and neuroinflammation; EGCG and resveratrol are promising, but clinical validation is still early.	Narrative clinical reviews, phase II studies, and a strong mechanistic pre-clinical base.	No robust and uniform aggregated clinical base; reviews describe small, short trials, including phase II studies in AD, PD, and memory.	Small samples, short duration, low bioavailability, and lack of confirmatory trials; in cancer, resveratrol effects can be ambiguous or even unfavorable in some contexts.	Low to moderate

* Data extraction was assisted by the Consensus artificial intelligence platform. The AI-generated initial search and synthesis were subsequently verified against the original peer-reviewed sources. All extracted data, clinical outcomes, and limitations were independently critically analyzed, validated, and finalized by the authors, who take full responsibility for the accuracy and integrity of the content.

However, the real clinical effectiveness of these interventions depends on bioavailability, food matrix, gut microbiota composition, and individual host characteristics [250].

Thus, the incorporation of foods naturally rich in BC should be strongly encouraged as part of healthy dietary patterns, while the use of isolated supplements or concentrated extracts should be based on consistent clinical evidence and individualized according to the patient’s context.

## 5. Translational Challenges of Bioactive Compounds

Plant-derived bioactive compounds—such as polyphenols, carotenoids, glucosinolates, and peptides—face shared translational barriers that compromise their clinical efficacy. These primary obstacles include limited oral bioavailability, the absence of standardized therapeutic dosages, and marked interindividual variability in metabolism and physiological response [9,24,251]. Low oral bioavailability remains the central bottleneck for most bioactive classes.

For instance, polyphenols and phytosterols regularly exhibit systemic bioavailability below 5–10%, severely restricting the concentration that reaches target tissues and organs [45]. Compounds such as curcumin, resveratrol, and epigallocatechin-3-gallate (EGCG) have poor absorption due to rapid first-pass metabolism in the intestinal wall and liver [234,252]. Furthermore, apigenin displays near-zero bioavailability due to its high hydrophobicity, low intestinal permeability, and rapid elimination kinetics [229]. A similar challenge limits flavonoids, whose clinical application remains restricted by poor water solubility and poor systemic absorption [253].

The food matrix plays a critical role in modulating this bioavailability. Processing techniques such as thermal treatments, mechanical homogenization, and fermentation can liberate carotenoids and polyphenols from the matrix, thereby enhancing intestinal uptake [9]. Similarly, the absorption of highly lipophilic compounds, including lycopene and lutein, is significantly improved through the co-ingestion of dietary lipids [9]. For glucosinolates and isothiocyanates, the food matrix can either potentiate or inhibit absorption pathways [24]. Conversely, excessive oxidation, prolonged heating, or light exposure degrade these bioactive molecules and compromise their chemical stability [9]. Advanced formulation strategies—such as nanoformulations, lipid-based encapsulation, and controlled-release delivery systems—aim to circumvent these pharmacokinetic limitations, though they inherently increase production costs and manufacturing complexity [252].

Interindividual variability (IIV) in metabolism and bioavailability represents one of the most consistently documented yet least characterized factors in bioactive translation. The gut microbiota plays a central role in this phenomenon by converting parent phenolics into active metabolites, serving as the primary driver of IIV across most polyphenol classes [141]. A systematic review of 153 studies identified that these variations between individuals stem primarily from the composition and metabolic activity of the gut microbiota, alongside genetic polymorphisms, age, sex, ethnicity, body mass index (BMI), and baseline physiological status [48]. A classic example is the ingestion of soy isoflavones: only about 30% of the Western population has a microbiota capable of synthesizing equol—a metabolite with potent estrogenic and cardiovascular protective activity—meaning these producers derive substantially greater cardiovascular benefits than non-producers [254].

Genetic profiles and single-nucleotide polymorphisms also profoundly influence clinical outcomes. Polymorphisms in genes encoding conjugative enzymes and transporters, such as UGT1A1, SULT1A1, and COMT, directly modulate the bioactivity of phenolic metabolites [141]. Similarly, genetic variants in carotenoid absorption and cleavage genes, including SR-BI, CD36, and BCO1, explain the distinct individual differences observed in systemic lutein responses [144,254]. Ultimately, overcoming clinical attrition and maximizing the therapeutic translation of bioactive compounds necessitates larger, highly standardized clinical trials. These studies must integrate validated biomarkers, optimized delivery vectors, and patient stratification based on individual metabotypes to resolve the interindividual variability that currently compromises reproducibility.

## 6. Limitations

Despite considerable advances in research on the BC in the diet, their immediate practical application in the clinic still faces the predominance of in vitro studies and animal models. Such models are often conducted with isolated concentrations that are markedly higher than the physiological fractions achieved through habitual feeding. In addition, the intrinsic differences in absorption, cleavage rate, and peripheral metabolism make it difficult to directly extrapolate these biological findings to human models in a linear fashion.

Another highly relevant aspect concerns the marked interindividual variability. Factors such as the gut microbiota profile, genetic polymorphisms, age, sex, basal nutritional status, concomitant medication use, and interactions with the food matrix directly influence the compound’s biological outcome. Added to this picture is the significant methodological heterogeneity observed among the published trials (variation in doses, intervention durations, and extract sources) and the relative scarcity of long-term controlled clinical trials with robust primary clinical outcomes. Therefore, more standardized, multicenter, and translational clinical studies are imperative to map the real therapeutic potential of these molecules adequately.

## 7. Conclusions

BC in the diet exerts biologically relevant effects on molecular networks associated with inflammation, redox balance, energy metabolism, and immune response, extrapolating the classic nutritional role of food.

Among the mechanisms most consistently described in contemporary literature, the coordinated modulation of signaling axes controlled by NF-κB, Nrf2/ARE, AMPK/mTOR, and the NLRP3 inflammasome stands out, directly impacting cellular homeostasis and attenuating chronic low-grade inflammation.

In this regulatory ecosystem, interactions with the gut microbiota play a central role. A bidirectional dynamic is established in which BC modify microbial composition and metabolic activity toward eubiosis. At the same time, the biotransformation performed by these microorganisms dictates the bioavailability and biological activity of metabolites circulating in the host. This coordinated interaction of the diet–microbiota–host axis is crucial for maintaining intestinal barrier integrity, stimulating the production of protective metabolites (such as SCFAs), and refining systemic immunometabolic regulation.

Together, these properties reinforce the strategic role of food in health promotion. However, for these scientific findings to support personalized clinical guidelines and evidence-based therapeutic recommendations in precision nutrition, more robust, controlled human clinical trials with properly standardized formulations are needed. In particular, such standardization should specifically address the food matrix (whole food versus isolated or purified compound), the intervention dose, interindividual differences in gut–microbiota composition, the intervention duration, and the demographic and clinical characteristics of the study population, as these variables directly determine the bioavailability, metabolic fate, and reproducibility of the biological effects attributed to dietary bioactive compounds.

## Data Availability

No new data were created or analyzed in this study. Data sharing is not applicable to this article.

## Abbreviations

The following abbreviations are used in this manuscript:

AMPK	AMP-activated protein kinase
BC	Bioactive compounds
COX-2	Cyclooxygenase-2
DHA	Docosahexaenoic acid
EGCG	Epigallocatechin gallate
EPA	Eicosapentaenoic acid
HO-1	Heme oxygenase-1
IBD	Inflammatory bowel disease
IL	Interleukin
iNOS	Inducible nitric oxide synthase
JAK/STAT	Janus kinase/signal transducer and activator of transcription
Keap1	Kelch-like ECH-associated protein 1
LPS	Lipopolysaccharide
MAPK	Mitogen-activated protein kinase
mTOR	Mechanistic target of rapamycin
NCDs	Non-communicable diseases
NF-κB	Nuclear factor kappa B
NLRP3	NOD-like receptor family pyrin domain-containing protein 3
NQO1	NAD(P)H quinone oxidoreductase 1
Nrf2	Nuclear factor erythroid 2-related factor 2
PPARs	Peroxisome proliferator-activated receptors
PUFA	Polyunsaturated fatty acids
ROS	Reactive oxygen species
SCFAs	Short-chain fatty acids
SIRT1	Sirtuin 1
T2DM	Type 2 diabetes mellitus
TNF	Tumor necrosis factor
VCAM-1	Vascular cell adhesion molecule-1
ω-3 PUFA	Omega-3 polyunsaturated fatty acids
